# Protective effect of walnut on d‐galactose‐induced aging mouse model

**DOI:** 10.1002/fsn3.907

**Published:** 2019-02-05

**Authors:** Ji Liu, Dan Chen, Zukun Wang, Chaoyin Chen, Delu Ning, Shenglan Zhao

**Affiliations:** ^1^ School of Chinese Materia Medica Yunnan University of Traditional Chinese Medicine Kunming China; ^2^ Yunnan Institute of Tobacco Quality Inspection and Supervision Kunming China; ^3^ Yunnan Academy of Forestry Kunming China

**Keywords:** cognitive function, d‐galactose, oxidative stress, walnut

## Abstract

**Objective (s):**

Accumulating evidence has suggested that oxidative stress and apoptosis are involved in the aging process. d‐galactose (gal) has been reported to cause symptoms of aging in mice, accompanied by liver and brain injuries. Our present work was to study the potential antioxidative and anti‐apoptotic effects of walnut and to explore how these effects act on mice in a d‐gal‐induced aging model.

**Materials and Methods:**

Aging mice were induced by subcutaneous injection of d‐gal (200 mg kg^−1^ d^−1^ for 8 weeks). Walnut samples were simultaneously administered to the d‐gal‐induced aging mice once daily by intragastric gavage. Finally, body weight, organ index, cognitive function, levels of antioxidative enzymes, and liver function were monitored.

**Results:**

The kernel pellicles of walnut could not only improve the learning and memory ability, and the organ index, but also significantly decrease body weight and normalize the levels of activity of antioxidative enzymes in aging mice. Further, the walnut seed coat would protect damages of hippocampus and liver in aging mice.

**Highlights:**

In the current study, we investigated the effects of walnut kernels and walnut seed coats (WSCs) on d‐galactose‐induced aging mice. WSC was firstly found to have beneficial effects on d‐gal‐treated mouse's brain with learning and memory impairment, which probably through the underlying mechanism reduces oxidative damage and limits neuroinflammation. In addition, WSC had a protective effect on liver damage in d‐galactose sensing mice.

## 1 INTRODUCTION

Aging is a major factor involved in neurological impairments, decreased antioxidant activities, and enhanced neuroinflammation (Rehman, Shah, Ali, Chung, & Kim, 2017). It is expected that by the year 2025, the elderly (over 65 years) population will exceed 800 million (Fatemi et al., 2018). Therefore, studying the pathophysiology of aging and related diseases is a significant challenge for medical gerontology (Kennedy & Pennypacker, 2014).

d‐galactose administration has been shown to induce impairments in memory and cognition in mice accompanied by aging‐associated deficits. The d‐gal exposure could also exacerbate oxidative damage, including increased content of malondialdehyde (MDA), and decreased total antioxidative capabilities (AOCs), total superoxide dismutase (SOD), and glutathione peroxidase (GSH‐Px) activities (Yin et al., 2010). Moreover, the d‐gal exposure could cause brain hippocampus and liver damage (Chen, Chen, & Zhou, 2018). Therefore, d‐galactose‐induced amnesia has been used widely for anti‐aging and organ injury studies (Chen et al., 2018; Lian et al., 2017).

Walnuts contain a number of antioxidant substances such as flavonoids, melatonin, vitamin E, and numerous polyphenols (Carvalho et al., 2010; Liang, Chen, Cao, & Zhao, 2017). The walnut seed coat (WSC) is rich in polyphenols (Shimoda et al., 2009), and the walnut seed (WS) mainly contains unsaturated fatty acids with antioxidative activity (Fukuda, Ito, & Yoshida, 2003; Li et al., 2006). The main chemical components of WSC and WS are shown in Table 1. Walnuts were reported to have antioxidant activity and could improve brain learning and memory functions (Shi et al., 2014). However, to our knowledge, there are neither reports to date about the protective effects of WSC and WS on livers and brains in d‐gal‐induced aging mice, nor reports regarding its underlying anti‐aging molecular mechanisms. Therefore, the purpose of this study was to evaluate the protective effects of WSC and WS on brain and liver of d‐galactose‐injected mice and to explore the possible mechanisms.

**Table 1:** Chemical characterization of walnut seed (WS) and walnut seed coat(WSC; 100 g dry weight)

	Total polyphenols (g)	Protein (g)	Crude fat (g)	Total sugar (g)
WS	0.77 ± 0.12	15.0 ± 1.21	60.0 ± 0.93	9.80 ± 0.61
WSC	37.64 ± 0.61	11.0 ± 0.89	20.2 ± 0.41	7.71 ± 0.98

*Note*

Data are presented as the mean ± *SD*.

## 2 MATERIALS AND METHODS

### 2.1 Reagents

Walnut seed coat and WS were cultivated in Zhaotong of Yunnan Province of China. WSC was crushed and dried at 30°C. WS was freeze‐dried at a ratio of 1:10 (g/ml) of juice. d‐gal was purchased from Sigma Chemical (purity ≧99%). Vitamin E (VE) was supplied by Meilun Biology Chemical Reagent Co., Ltd. (Shanghai, China, purity >98%).

Assay kits for the measurements of aspartate aminotransferase (AST, No. 20180734), alanine aminotransferase (ALT, No. 20180531), MDA (No. 201805 56), and SOD (No. 20180432) were obtained from Nanjing Jiancheng Biological Engineering Research Center (Nanjing, China). All the other solvents and reagents used in the study were at least of analytical grade.

### 2.2 Animals and treatments

All treatment and maintenance of animals were performed in accordance with the Animal Care Committee of Peking Union Medical College and Chinese Academy of Medical Sciences (Beijing, China).

Male KM mice (18–20 g, 8 weeks) were purchased from Silaike Jingda Experimental Animal Co., Ltd. (Hunan, China; Animal certificate number: SCXK‐(Hunan) 2018‐002). Mice had free access to food and water under‐housed in an air‐conditioned room (20–26°C, 40%–70% RH) with a 12‐hr/12‐hr light–dark cycle. After 1 week of acclimatization, the mice were divided randomly into five groups (*n* = 10 each): the blank control group, the model group (d‐gal), the positive control group (VE, 100 mg/kg), the walnut seed‐treated group (WS, 600 mg/kg), and the walnut seed coat‐treated group (WSC, 100 mg/kg). Except for the blank control group, all groups were subcutaneously injected d‐gal (200 mg kg^−1^ d^−1^) dissolved in normal saline solution (0.9%, *w/v*) for 8 weeks. The mice in the WSC, WS, and VE groups (Tween 80 was used to dissolve VE) were orally gavaged, while the mice in the control group were treated with hypodermic injections of 0.9% normal saline (100 mg/kg) at equal volumes and concomitantly administered normal saline by gavaged every day. All animals were regularly monitored for weight on a weekly basis, and the dose was adjusted accordingly.

### 2.3 Determination of body weight and organ index

At the end of the experiment, mice were euthanized. Fresh brain and liver tissue were obtained for biochemical measurements and histopathological determination. The spleen, thymus, kidneys, and spleen were separated from the body and then weighed to calculate the organ index coefficients. The animal organ index calculation method is defined as the coefficient (mg/g) = organ weight (mg)/body weight (g).

### 2.4 Behavioral tests

#### 2.4.1 Autonomous activity test

Autonomous activity testing was performed before all behavioral tests (Ennaceur & Delacour, 1988). The device consisted of a squared open black box that was evenly divided into 25 squares and a capture system that recorded the trajectory. A mouse was put into the autonomous activity box, and the number of grooming in 5 min was recorded.

#### 2.4.2 The novel object recognition task

The experiment was conducted in a self‐made open black plastic box (50 × 40 × 30 cm), which mainly consisted of three phases (Yang et al., 2015). During the first phase of habituation, the mouse was allowed to freely explore the empty box for 5 min. Twenty‐four hours after habituation, the mouse was pretested and exposed to the familiar empty box for 2 min followed by an interval of 2 min before the second phase of object sampling experiment. In this phase of experiment, the mouse was allowed to explore the box arena with two identical objects (A, B, blue solid glass cylinder with a height of 10 cm and a bottom diameter of 5) for 5 min. After 24 hr, a selective test was performed for the last phase of object recognition experiment. At this time, one of the explored objects was replaced in situ by a new one (e.g., A or B was replaced by C, and the object C was a colored plastic building block each having a length, width, and height of 4 cm). The position of another object was constant, allowing the mouse to freely explore for 5 min. After each mouse was explored, the device and the experimental object were thoroughly cleaned, first scrubbed with 75% alcohol, rinsed with water, and dried with new towel for use. Results were expressed as discrimination ratio (the difference between exploration time of novel and familiar divided by the total time spent exploring the objects in the test phase). The time of exploration began with the mouse's nose at a distance of ≤1 cm or when the mouse touches things directly with the nose (counting when the mouse climbs on or moves around the object).

#### 2.4.3 Step‐down test

The experiment was completed in 2 days (Zhou et al., 2016). On the first day, the mice were placed in a platform jumper to adapt and habituate the environment for 120 s, and then a 2‐mA current stimulation was given for 180 s. When the mice were stimulated by electricity, they jumped onto the safety platform. The time of the first jumping was the incubation period. Incubation period <120 s was recorded. If the mouse did not jump off the platform, the incubation period was calculated as 3 min, and in addition, the total number of electric shocks the mice received after the incubation period was recorded. The above operation was repeated after 24 hr.

### 2.5 Organization collection and testing

The mice organ (liver, brain) segments (approximately 200 mg) were homogenized in 2,000 μl of chilled normal saline. After centrifugation (4,000 *g*, 10 min, 4°C) of the homogenates, the supernatants were transferred to the other test tubes. ALT and AST levels in livers, and SOD, MDA, AChE, and ATPase levels in brain tissues were detected by the kit instructions. (All kits are provided by Nanjing Jiancheng Bioengineering Institute.)

### 2.6 Histological study

Three mice from each group were selected for HE staining of the hippocampus and liver tissues.

### 2.7 Statistical analysis

Behavioral test data were expressed as the mean ± *SEM*. Other data were expressed as the mean ± *SD*. Statistical analysis was conducted in GraphPad Prism 7.0 software. Data were evaluated with one‐way ANOVA following the Dunnett's multiple comparisons test at a 95% confidence level.

## 3 RESULT

### 3.1 Body weight and organ index

As shown in Table 2, after the eighth week, the d‐gal model group had a significant decrease in body weight compared with the control group (*p* < 0.05), and the remaining administration groups reversed d‐gal‐induced weight loss (*p* < 0.05). The brain index, liver index, and thymus index of the model group were decreased significantly compared with the blank control group (*p* < 0.05). But the administration of VE or WSC could increase brain index and thymus index compared to the d‐gal‐induced aging group (*p* < 0.05 or <0.01).

**Table 2:** Effects of walnut seed coat (WSC) and walnut seed (WS) on body weight and organ index in d‐gal‐treated mice

Group	Body weight (g)	Organ index (mg/g)				
		Brain index	Liver index	Thymus index	Heart index	Spleen index
Blank control	44.84 ± 2.56	1.22 ± 0.09	5.94 ± 0.46	0.15 ± 0.03	0.54 ± 0.05	0.31 ± 0.03
d‐gal	38.25 ± 1.06###	1.05 ± 0.02##	4.77 ± 0.48###	0.08 ± 0.02##	0.55 ± 0.07	0.29 ± 0.04
VE (100 mg/kg)	42.77 ± 4.03*	1.19 ± 0.04*	5.46 ± 0.30*	0.14 ± 0.02**	0.54 ± 0.04	0.31 ± 0.05
WS (600 mg/kg)	42.93 ± 1.36*	1.11 ± 0.08	5.36 ± 0.27	0.15 ± 0.03**	0.53 ± 0.03	0.30 ± 0.04
WSC (100 mg/kg)	42.76 ± 2.84*	1.19 ± 0.05*	5.10 ± 0.52	0.13 ± 0.03*	0.59 ± 0.03	0.28 ± 0.06

*Notes*

VE: vitamin E.

Values were expressed as mean ± *SD* (*n* = 10 in each group).

^##^ *p* < 0.01 and ^###^ *p* < 0.001 compared to the control group.

*p < 0.05 and **p < 0.01 compared to the d‐gal group.

### 3.2 Effects of WSC on memory and cognition of d‐galactose‐induced aging mice

#### 3.2.1 Effect on autonomous activity

**Figure 1:**
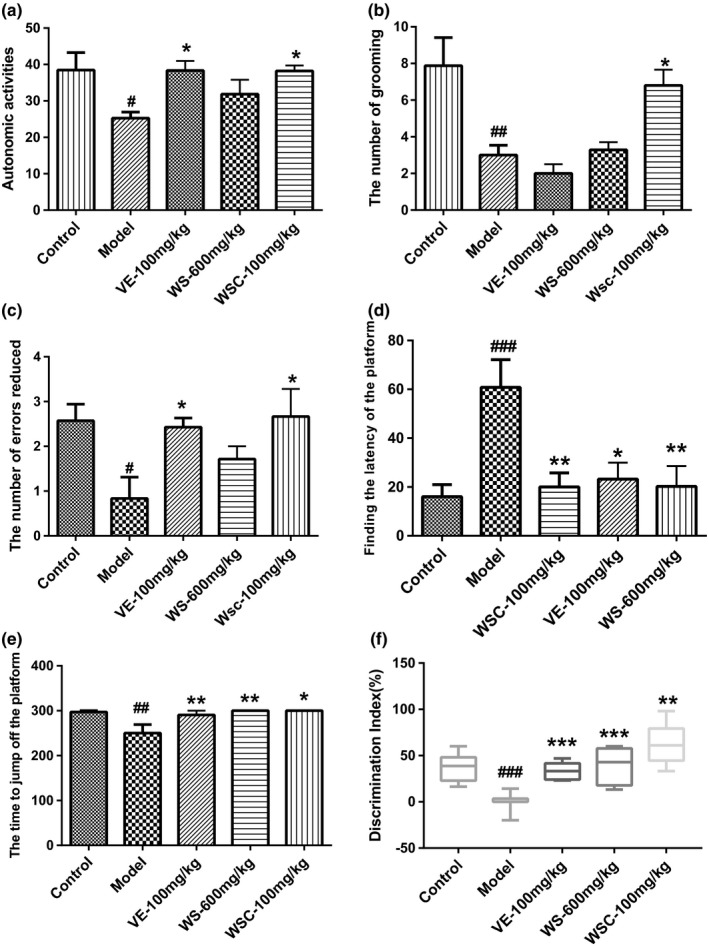
Walnut seed coast (WSC) improves Memory and Cognition in D‐galactose Administered Mice in Behavioral Tests. Data were expressed as mean ± SEM (n = 8‐10). (A‐B) The effect of WSC was on the performance of mice in the autonomous activity test. (A) Compared with model group long‐term administration of WSC had enhanced the autonomous activities (*p*<0.05). (B) WSC treatment can significantly increase the number of hairs in mice (*p*<0.05). (C‐E) The effect of WSC was on the performance of mice in the step‐down test. (C) WSC and VE could prolong the number of errors reduced (*p*<0.05). (D) WSC, VE, WS have reduced the finding the latency of the platform times (*p*<0.05 for all). (E) WSC, VE, WS could prolong the time to jump off the platform (*p*<0.05 for all). (F) Treatments with WSC, WS, and VE prevented D‐gal‐induced learning and memory deficits and their novelty preference (*p*<0.01 for all). ^#^ *p*< 0.05, ^##^ *p*<0.01, ^###^ *p*<0.001 vs. control group. *p<0.05, **p<0.01, ***p<0.001 vs. model group

Autonomous activities of mice were evaluated before other behavioral tests to exclude the effect of reagents (d‐galactose, WSC, WS, VE) on motor function. As in Figures 1a, compared with the d‐gal model group, the long‐term administration of WSC had enhanced the autonomous activities (*p* < 0.05). At the same time, WSC treatment had significantly increased the number of hairs in mice (Figure 1b, *p* < 0.05).

**Figure 2:**
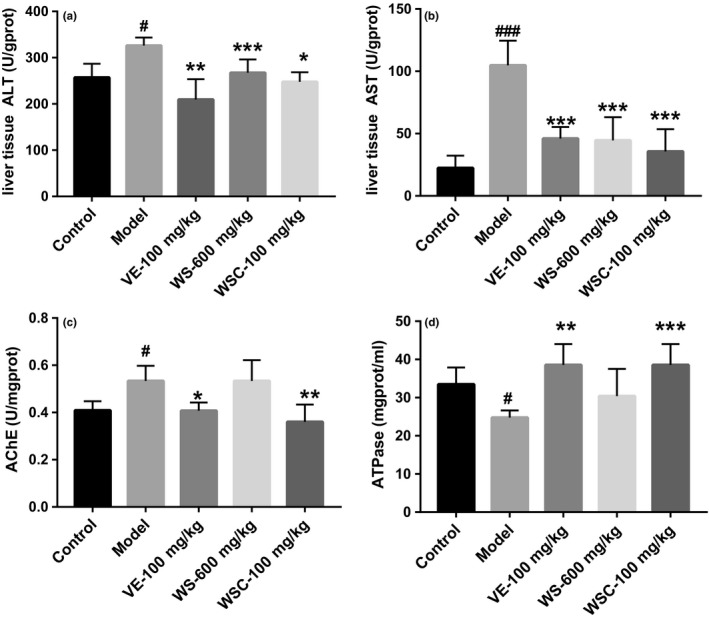
Walnut seed coat (WSC) tended to increase ATPase levels and decrease AChE activity in the brain and decrease liver aspartate aminotransferase (ALT), alanine aminotransferase (AST). Data were expressed as mean ± SD (n = 8‐10). (A‐B) WSC, WS, VE would decrease the AST and ALT level in D‐gal treated mice (*p*<0.05 for all). (C) WSC and VE tended to inhibit AChE activity. (D) The level of ATPase in the brain of D‐gal treated mice was significantly lower than that of the controls (*p*<0.05). ^#^ *p*<0.05, ^###^ *p*<0.001, vs. control group, *p<0.05, **p<0.01, ***p<0.001 vs. model group

#### 3.2.2 Effect on the novel object recognition

The working memory of d‐gal mice was impaired of which the recognition index was significantly lower than other groups (*p* < 0.01). Treatments with WSC, WS, and VE had prevented d‐gal‐induced learning and memory deficits and improved their novelty cognitive ability (Figure 1f).

#### 3.2.3 Effect on step‐down test

The step‐down test is a type of passive avoidance task in which the latency time and the error number are the two main indices. As shown in Figure 1c–e, for the d‐gal model group, the number of errors reduced and the time to jump off the platform were significantly declined (*p* < 0.05), and corresponding finding the latency of the platform had increased significantly (*p* < 0.01). In comparison with the model group, the WSC and VE groups could prolong the number of errors reduced and the time to jump off the platform (*p* < 0.05 for all), and reduce the finding the latency of the platform times (*p* < 0.05 for all).

### 3.3 Effects of WSC on ATPase and AChE in the brain and liver ALT and AST

As shown in Figure 2a–b, the AST and ALT levels were significantly increased in d‐gal group compared to the control group (*p* < 0.05). In contrast, WSC, WS, and VE administrations would decrease the AST and ALT levels in d‐gal‐treated mice (*p* < 0.05 for all). WSC and VE intended to restore d‐gal‐induced aging impairments by decreasing ATPase level (*p* < 0.05 for all) and inhibiting AChE activity, while WS had no effect on these two indices (*p* < 0.05 for all; Figure 2c). The level of ATPase in the brain of d‐gal‐treated mice was significantly lower than that in control group (*p* < 0.05; Figure 2d).

**Figure 3:**
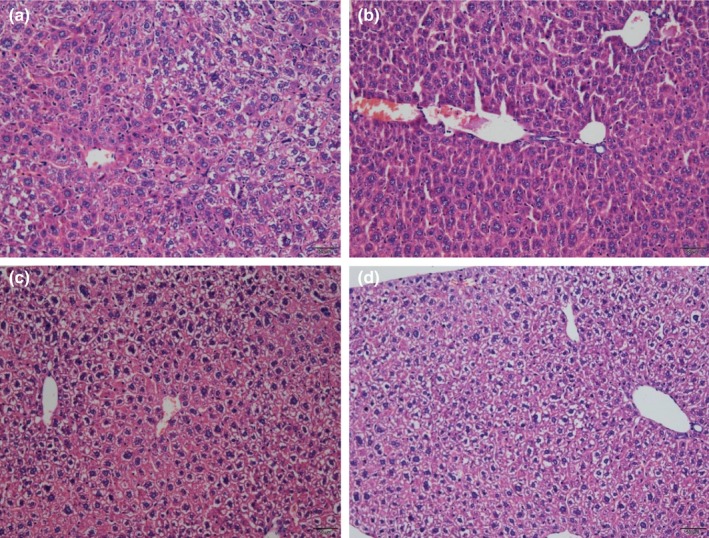
Effect of walnut seed coat (WSC) and walnut seed (WS) were treatment on liver (H & Estaining, magnification 40×). (a) Blank control group; (b) D‐gal model group; (c) D gal+VE100mg/kg; (d) D‐gal+WSC100mg/kg

### 3.4 Protects of WSC on hippocampus and liver harm from d‐galactose mice

As observed visually in Figure 3b, the histological analysis showed that d‐gal injection resulted in moderate levels of hepatocyte apoptosis, necrosis, and inflammatory cell infiltration. However, WSC treatment had shown a great protective effect, which had significantly alleviated the pathological changes in the liver. The morphological structure of WSC group was almost the same as that of VE and blank control group.

**Figure 4:**
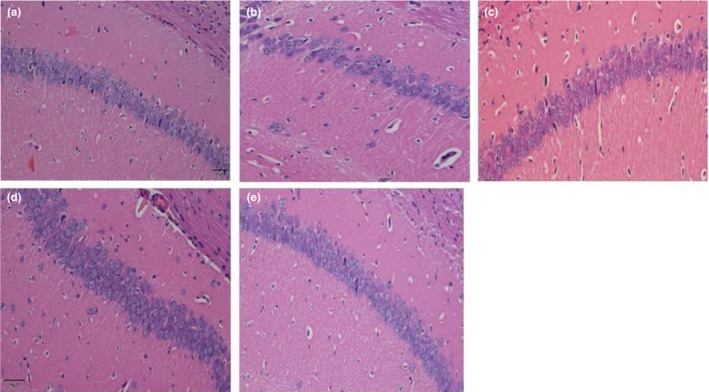
Effect of walnut seed coat (WSC) and walnut seed (WS) were treatment on CA1 region of hippocampus (H&Estaining, magnification 40×). (a) Blank control group; (b) D‐gal model group; (c) D‐gal+VE100mg/kg; (d) D‐gal+WS600mg/kg; (e) D‐gal+WSC100mg/kg

As seen in Figure 4b, in the d‐gal model group, the hippocampal neurons were arranged disorderly with a significantly reduced cell number. However, there was an obvious increase in round‐shaped neurons and well‐organized fibers after the intervention of WSC. The morphological structure of WSC treatment was very similar to that of the blank control group and the positive control group (Figure 4c), indicating that the degree of pathological changes in brain tissue would be mitigated by WSC treatment.

## 4 DISCUSSION

In this study, we used a d‐galactose‐induced amnesic model to evaluate the therapeutic effect of WSC and WS on the cognitive dysfunction caused by aging. In addition, we had studied the protective effect of WSC on hippocampus and liver injury in d‐galactose mice. d‐galactose‐induced memory loss associated with aging was a well‐recognized classic model. Its neurotoxicity, induced by abnormal accumulation of ROS and AGEs, has been widely reported (Baeta‐Corral, Castro‐Fuentes, & Gimenez‐LLORT, 2018). In previous studies, it had been demonstrated that oxidative stress might lead to apoptosis, which was the main cause of many types of organ damages. Previous studies also showed that mitochondrial ROS induced the activation of a large number of mitochondrial apoptotic proteins, leading to the cellular apoptosis and organ damages (Chen et al., 2018). d‐galactose‐induced aging mouse's liver and brain hippocampi were more susceptible to be injured (Rathod, Kale, & Joshi, 2016). Using this model to study the effects of supplements and antioxidants has the potential to proposed new therapies for liver and brain damage due to aging.

Walnut seed coat is rich in polyphenolic compounds (Shimoda et al., 2009). WS is rich in unsaturated fatty acids (Ito, Okuda, Fukuda, Hatano, & Yoshida, 2007). Many studies have shown that they all have a good antioxidant activity (Fukuda et al., 2003; Li et al., 2006). In our study, we aimed to demonstrate that during a period of 8 weeks of aging model establishment via subcutaneous injection of d‐gal in mice, WSC and WS could protect against d‐gal‐induced organ injury in vivo. Studies showed that mice subcutaneously injected the d‐gal neck exhibited a significant memory loss, body weight declined, and organ index reduced (Baeta‐Corral et al., 2018; Chen et al., 2018; Zhang et al., 2018). In present studies, it had been clearly demonstrated that the administration of d‐gal had caused the atrophy of the brain, thymus, and liver, while no significant changes were found on spleen and hear. In addition, d‐galactose was found to significantly reduce body weight in mice; however, WSC would improve organ index and weight loss.

Mice had been subjected to 8‐week injection of d‐galactose (200 mg/kg, s. c.) and treatment of drugs before the behavioral tests were conducted. This study used a series of tests to fully evaluate the memory and cognitive function. In the autonomous activity test, the motor function of mice was different. Movement distance and movement path in the middle region could infer the mouse's cognitive and exploration capabilities in the mine experiment. Under normal circumstances, animals would actively explore the new environment, but animals with poor cognitive ability would have less activity in the new environment (Yamamuro, Yoshimura, Tsuchiya, Sensui, & Asou, 2004). The grooming behavior of animals appeared in a more comfortable environment and a leisure activity in a new environment, indicating that mice were more adaptable in an unfamiliar environment (Zhang et al., 2017). WSC treatment had enhanced the autonomous activities and increased the number of hairs in mice, which indicates that WSC treatment would enhance the excitability and environmental adaptability of d‐galactose‐induced senile mice. The step‐down experiment mainly examined the relevant mouse's associative learning capabilities (Huang et al., 2001). Our results showed that d‐galactose injection could significantly increase the number of errors in a mouse's platform trial and significantly increase the time to jump off the platform. In this experiment, WSC treatment had remarkably improved the learning performance, for instance, reduced the number of errors and the time to jump off the platform significantly (*p* < 0.05), and increased the finding the latency of the platform significantly (*p* < 0.01). In the novel object recognition task, we observed a significant difference in NORT results between control and d‐gal‐treated mice. This showed that long‐term administration of d‐gal for 8 weeks led to a novelty–cognition impairment of exploration behavior. Our results showed that WSC, VE, and WS had improved the working memory and novelty preference of d‐gal‐treated mice. However, WSC was more effective than other medications. In short, WSC could improve the memory and cognition of d‐galactose‐induced aging mice in behavioral tests.

d‐gal injections had been reported to cause liver damage and dysfunction, which would lead to an overall morphological change, as well as increase levels or activities of some serum enzymes (Li et al., 2016). This study showed that ALT and AST levels were significantly elevated in mouse livers injected with d‐gal for 8 weeks. In addition, WSC and WS would significantly reduce liver ALT and AST levels. Cholinergic systems were widely involved in synaptic connections and formed the basis of information transmission (Schliebs & Arendt, 2006). Cholinergic system dysfunction was the basis of memory and cognitive deficits. In this experiment, d‐galactose increased AChE activity in mouse brain, which had mimicked the cholinergic dysfunction. The WSC (100 mg/kg) had inhibited AChE activity in d‐gal mouse model, which inferred that WSC would improve learning and memory function by exerting obstacle to inhibit the activity of cholinesterase. The energy supply provided by brain ATPase played an important role in the transmission of neuron information. Insufficient supply of ATP in brain tissue often led to the decreased sensitivity and activity of neurons. This study found that WSC (100 mg/kg) treatment would increase ATPase activity in d‐gal mouse model. In addition, we also examined hepatocyte apoptosis and inflammatory cell infiltration in liver tissues and recorded the number and arrangement of neuronal cells in the hippocampal CA1 area (Lakshmi, Sudhakar, & Prakash, 2015). After WSC treatment, the histopathological changes in the liver and brain were improved, indicating that WSC had a protective effect on mouse liver and brain during d‐gal‐induced aging.

## 5 CONCLUSION

We investigated whether WSC and WS could improve the liver and brain damage and the learning and memory impairment in mice induced by d‐gal. We found WSC and WS could alleviate liver and brain damages due to aging. Further, WSC used as the therapeutic was rich in polyphenols and had a better protection against liver and brain damages and learning and memory impairment in d‐galactose‐induced aging mice. Its mechanism might involve normalization of acetylcholinesterase activity, improvement of histopathological changes, and supply of ATP energy in brain tissue. However, the specific molecular mechanism of its role remains to be explored.

## CONFLICT OF INTEREST

The authors have no conflict of interest with others.

## ETHICAL STATEMENTS

This study has not any potential sources of conflict of interest. All animals were housed and cared for in accordance with the Chinese Pharmacological Society Guidelines for Animal Use.
